# Supplementary material for: Complexome profiling of the Chlamydomonas psb28 mutant reveals TEF5 as an early PSII assembly factor
Source: Plant Cell. 2025 Mar 17;37(6):koaf055. doi: 10.1093/plcell/koaf055 (PMC12164590; doi:10.1093/plcell/koaf055)
Supplement: koaf055_Supplementary_Data [file koaf055_supplementary_data.zip › psb28_interactive.zip!/plots/intensity/qualityPlots/peptides/_Cre06.g300550.t1.2.html]

### Cre06.g300550.t1.2

Trivial name:   
SeqCoverage [median]: 10.70  
Mass: 105.68 kDa  
  
 

| Sequence | MissedCleavage | Mass [Da] | wtIntensity | muIntensity | Score |
| --- | --- | --- | --- | --- | --- |
| AAGGAEQPAGPR | 0 | 1080.53 | 846.41 | 1358.70 | 170.69 |
| ADVNKLYDR | 1 | 1092.56 | 0.00 | 379.00 | 46.33 |
| AMSPQDKAMDR | 1 | 1248.56 | 0.00 | 393.05 | 55.40 |
| EAEDDRLAR | 1 | 1073.51 | 508.06 | 1995.40 | 69.81 |
| FKPYGEVGLR | 1 | 1164.63 | 1381.41 | 2957.98 | 99.97 |
| GVDPVEAYRR | 1 | 1160.59 | 1267.96 | 2503.21 | 64.04 |
| LYEQAGVSKEDER | 1 | 1522.73 | 1167.30 | 3569.67 | 76.36 |
| LYEQAGVSKEDERR | 2 | 1678.83 | 0.00 | 449.08 | 53.25 |
| VAHNYAAIYR | 0 | 1176.60 | 695.97 | 3628.11 | 63.22 |
| YLAAADEVLR | 0 | 1119.59 | 514.89 | 1921.51 | 90.66 |
|  |  |  | 6382.0 | 19155.7 |  |
